# Cholesterol Crystal Embolism Following Mechanical Thrombectomy for Acute Ischemic Stroke

**DOI:** 10.7759/cureus.72022

**Published:** 2024-10-21

**Authors:** May Pyae Kyaw, Tatsuya Tanaka, Jun Ito, Ryosuke Matsuoka, Mina Komuta, Akira Matsuno

**Affiliations:** 1 Department of Neurosurgery, International University of Health and Welfare Narita Hospital, Narita, JPN; 2 Department of Nephrology, International University of Health and Welfare Narita Hospital, Narita, JPN; 3 Department of Anatomic Pathology, International University of Health and Welfare Narita Hospital, Narita, JPN

**Keywords:** acute ischemic stroke, acute kidney injury, atheroembolic stroke, blue toe syndrome, cholesterol crystal embolism, endovascular complications, mechanical thrombectomy, skin biopsy

## Abstract

Cholesterol crystal embolism (CCE) is a rare but serious complication of atherosclerotic plaque rupture, often occurring after endovascular interventions. We report the case of a 73-year-old man who developed CCE following mechanical thrombectomy (MT) for an acute ischemic stroke (AIS) due to left internal carotid artery occlusion. The patient, with a history of hypertension and hyperlipidemia, underwent successful MT with complete recanalization. However, four weeks after the procedure, he presented with blue toe syndrome and acute kidney injury. Histopathological analysis of a skin biopsy confirmed the diagnosis of CCE. The patient’s renal function partially improved with corticosteroid therapy. Clinicians should maintain a high level of suspicion for CCE in patients who develop renal dysfunction or peripheral ischemia after MT, particularly in those with significant atherosclerotic risk factors. Early detection and timely intervention are critical for improving outcomes. Further research is warranted to clarify the incidence and optimal management of CCE in the context of MT for AIS.

## Introduction

Cholesterol crystal embolism (CCE) is a condition characterized by the embolization of cholesterol crystals from atherosclerotic plaques in large vessels, such as the aorta, to distal sites [1,2]. This phenomenon can lead to peripheral arterial occlusion and trigger inflammatory responses, potentially resulting in multiorgan failure, with acute renal failure being the most common manifestation [3,4].

Cholesterol crystal embolism can occur as a complication following endovascular procedures [1,2,5-11]. Risk factors for developing CCE include direct vascular injury, anticoagulant therapy, and thrombolytic therapy [1,2,8,9].

In recent years, mechanical thrombectomy (MT) has markedly improved outcomes for patients with acute ischemic stroke (AIS) due to large vessel occlusion, leading to its widespread use [12]. Cholesterol crystals have been observed in thrombi retrieved during MT, and some patients have been diagnosed with cerebral embolism secondary to aortic atherosclerosis-related cholesterol embolism [13-15]. However, to our knowledge, no cases of CCE occurring after MT have been reported. Herein, we present a case of CCE manifesting as blue toe syndrome and acute kidney injury following MT for AIS.

## Case presentation

**Figure 1:**
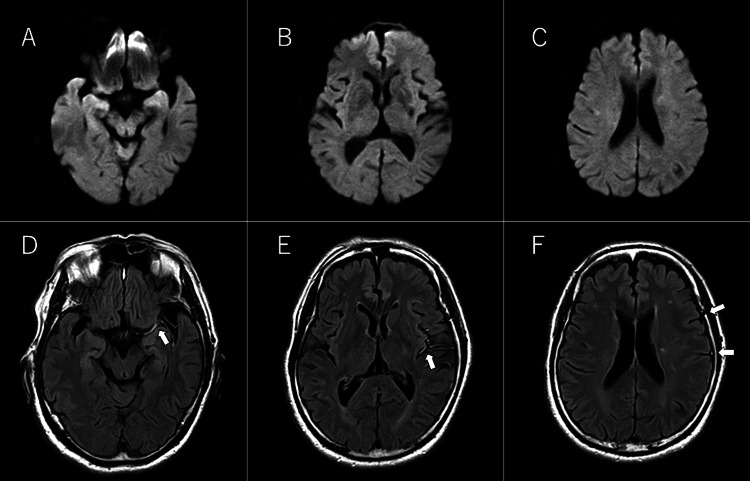
The patient's initial magnetic resonance imaging No ischemic changes were detected on diffusion-weighted imaging (A–C). Fluid-attenuated inversion recovery imaging revealed a hyperintense vessel sign in the left middle cerebral artery (D–F, arrow).

A 73-year-old man presented to our hospital with right-sided hemiplegia and impaired consciousness 47 minutes after onset. The patient’s medical history included hypertension and hyperlipidemia. The patient had a smoking history of 20 cigarettes per day for 53 years and consumed alcohol daily (20 g/day). Upon admission, the patient’s vital signs were as follows: heart rate, 83 beats/min; respiratory rate, 16 breaths/min; blood pressure, 145/83 mmHg; and SpO2, 100%. Neurological examination revealed a Glasgow Coma Scale score of 10: E3V2M5, left gaze deviation, right-sided hemiplegia, and mixed aphasia. Given the sudden onset of hemiplegia with cortical signs, a stroke was suspected. The National Institutes of Health Stroke Scale score was 16. Laboratory tests revealed N-terminal prohormone of brain natriuretic peptide of 132 pg/mL, D-dimer of 0.58 μg/mL, blood urea nitrogen (BUN) of 22.3 mg/dL, creatinine (Cre) of 1.34 mg/dL, and an estimated glomerular filtration rate (eGFR) of 41.1 mL/min/1.73 m^2^. The electrocardiogram did not show atrial fibrillation. Magnetic resonance imaging (MRI), performed 89 minutes after onset, revealed no ischemic changes on diffusion-weighted imaging (DWI); however, a hyperintense vessel sign was observed in the left middle cerebral artery (MCA) on fluid-attenuated inversion recovery imaging (Figure 1).

**Figure 2:**
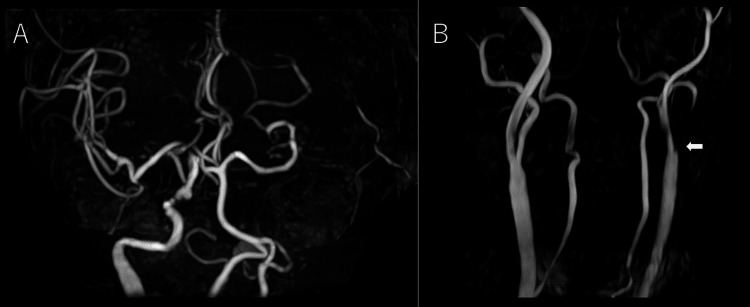
Cerebral and cervical magnetic resonance angiography Cerebral magnetic resonance angiography (MRA) (A) and cervical MRA (B) demonstrated occlusion of the left internal carotid artery (B, arrow).

Magnetic resonance angiography (MRA) revealed occlusion of the left internal carotid artery (ICA) (Figure 2).

**Figure 3:**
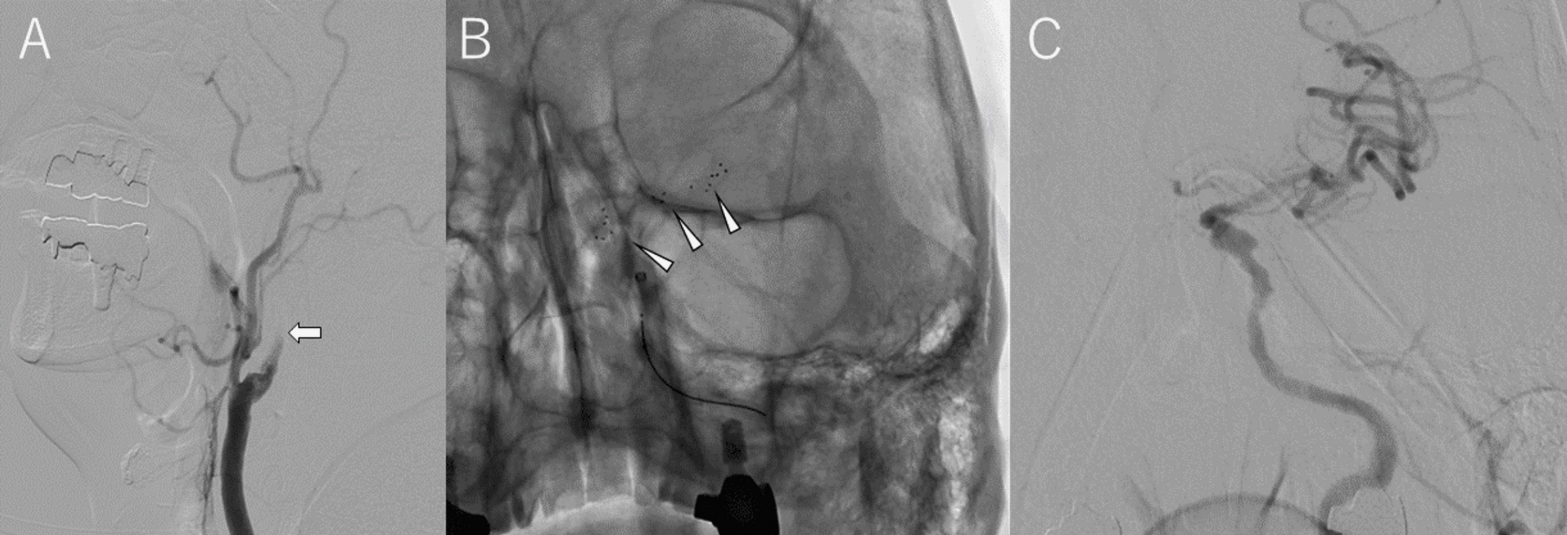
Mechanical thrombectomy Digital subtraction angiography (DSA) confirmed occlusion of the left internal carotid artery (A, arrow). A radiograph showed stent deployment from the left middle cerebral artery to the internal carotid artery (B, arrowhead). Complete recanalization of both the internal carotid artery and middle cerebral artery was confirmed on follow-up DSA (C).

The patient was diagnosed with AIS caused by a large vessel occlusion. Alteplase was intravenously administered 132 min after symptom onset. Mechanical thrombectomy was initiated 179 min after symptom onset. An 8-French OPTIMO balloon-guiding catheter (Tokai Medical Products, Kasugai, Japan) was placed in the cervical portion of the left ICA using a 5-French SIM catheter (Cook Medical, Bloomington, IN, USA) as an inner catheter. Cerebral angiography confirmed left ICA occlusion (Figure 3A). A 6-French Catalyst 6 aspiration catheter (Stryker, Portage, MI, USA), Trevo Trak21 microcatheter (Stryker), and CHIKAI 1014 microguidewire (Asahi Intecc, Seto, Japan) were used to advance the microcatheter beyond the occlusion. A 6 × 40 mm Solitaire stent retriever (Medtronic, Minneapolis, MN, USA) was deployed from the left MCA to the ICA (Figure 3B). Thrombus removal was achieved through simultaneous aspiration and stent retrieval, resulting in complete recanalization of the ICA and MCA within 18 minutes of treatment initiation (thrombolysis in cerebral infarction grade 3) (Figure 3).

**Figure 4:**
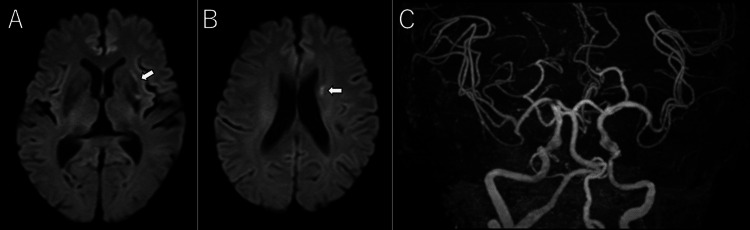
Post-mechanical thrombectomy magnetic resonance imaging and angiography Diffusion-weighted imaging demonstrated small acute infarcts in the left putamen and corona radiata (A, B, arrowhead). Magnetic resonance angiography showed no evidence of residual vascular occlusion (C).

A red thrombus was observed, but histological analysis was not performed. Post-MT MRI revealed hyperintense signals in the left putamen and corona radiata on DWI. The MRA did not show any vascular occlusions (Figure 4).

Carotid ultrasonography, cardiac ultrasonography, and Holter electrocardiography did not detect any embolic sources. Consequently, the patient was diagnosed with atherothrombotic cerebral embolism. Treatment included aspirin (100 mg/day), clopidogrel (75 mg/day), and rosuvastatin (5 mg/day). The patient’s hemiparesis and aphasia improved, and the patient was discharged on the 11th day with a modified Rankin Scale score of 0.

**Figure 5:**
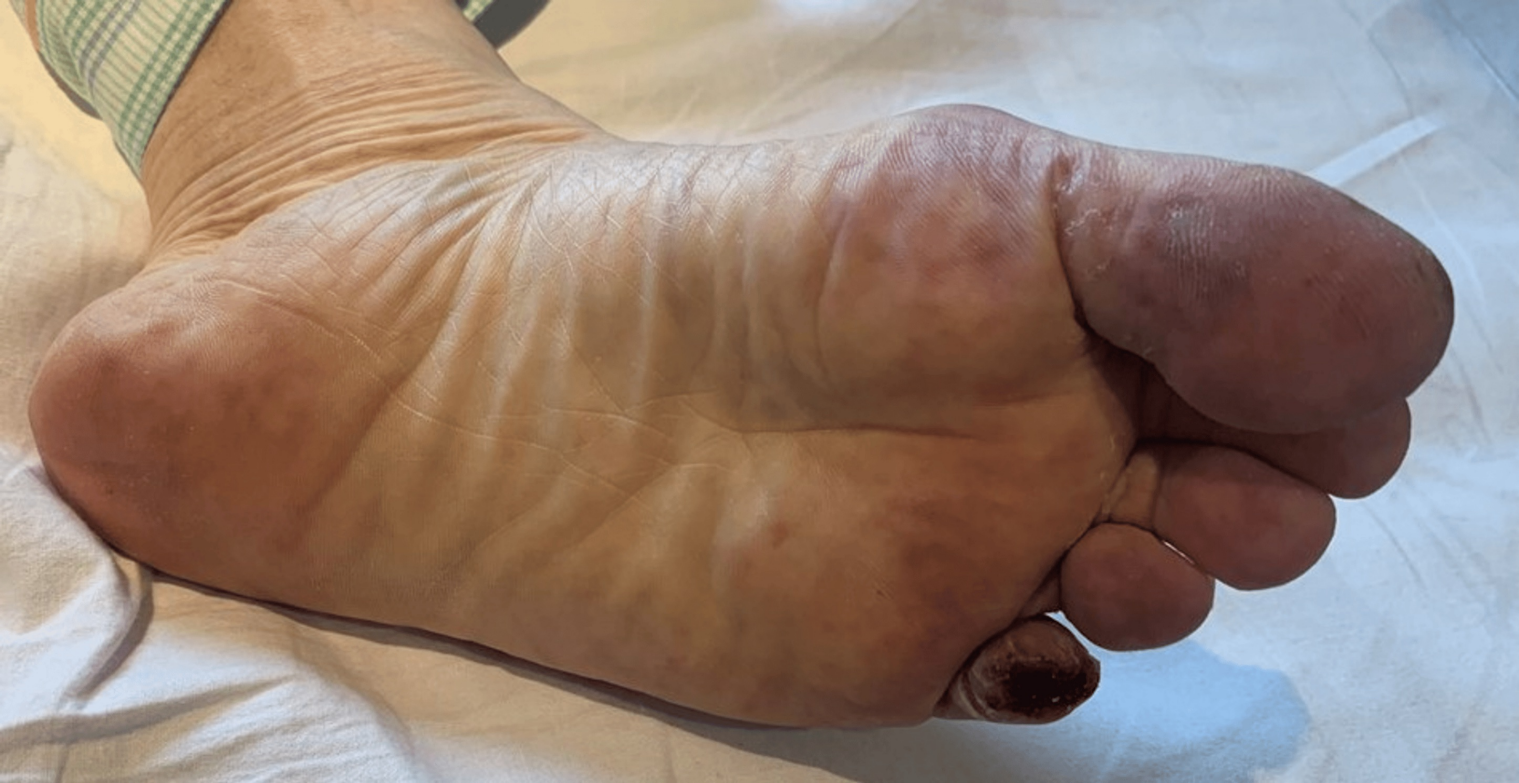
Photograph of the patient's left foot Multiple purpuric lesions, livedo reticularis, and blue toe syndrome were observed on the distal plantar surface and toes of the left lower extremity.

Four weeks after MT, the patient developed discoloration of the left fifth toe and leg pain and returned to our hospital five weeks after MT. During this time, the patient’s vital signs were as follows: heart rate, 92 beats/min; blood pressure, 150/109 mmHg; and SpO2, 98%. The dorsalis pedis artery was palpable. Numerous purpuric lesions, livedo reticularis, and blue toe syndrome were observed on the distal plantar surface and toes of the left lower limb (Figure 5).

**Figure 6:**
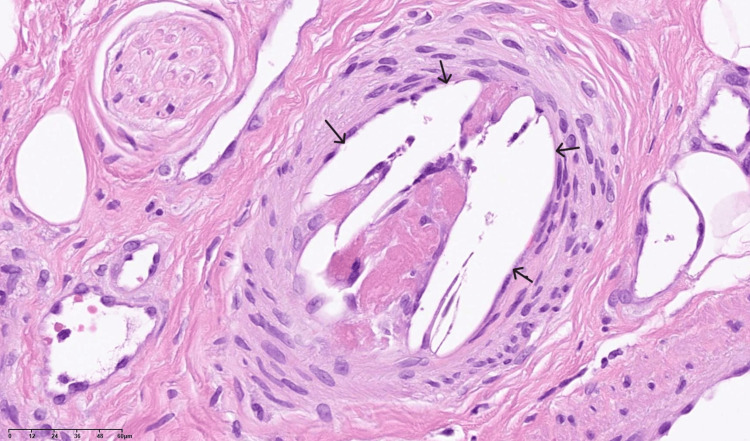
Pathological findings from the skin biopsy A histopathological section (hematoxylin and eosin staining) of a skin biopsy specimen revealed thrombotic occlusion and the characteristic cholesterol crystal clefts within an arteriole (arrow) (scale bar: 60 μm).

Laboratory tests revealed a BUN level of 65.9 mg/dL, Cre level of 4.35 mg/dL, and eGFR of 11.3 mL/min/1.73 m2, indicating acute kidney injury. Considering the onset of renal dysfunction and skin manifestations following endovascular intervention, cholesterol embolism was suspected. To confirm the diagnosis, a skin biopsy of the left fifth toe was performed, which revealed cholesterol crystal emboli within the vasculature of the deep adipose tissue (Figure 6).

**Figure 7:**
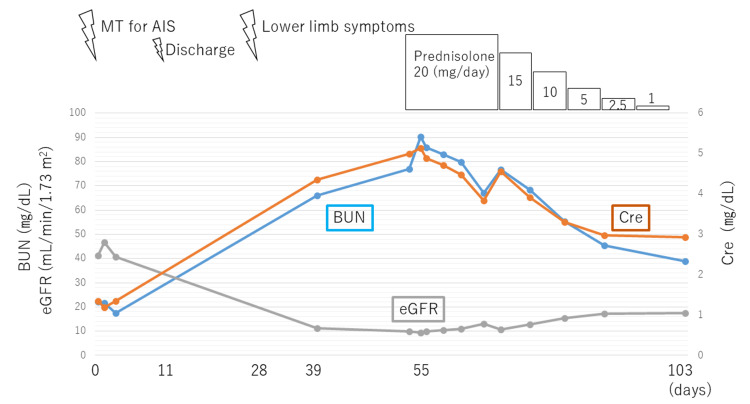
The patient's clinical course After the definitive diagnosis of cholesterol crystal embolism, oral administration of 20 mg prednisolone was initiated. Renal function gradually improved, and the prednisolone dose was tapered. Abbreviations: MT: mechanical thrombectomy; AIS: acute ischemic stroke; BUN: blood urea nitrogen; Cre: creatinine; eGFR: estimated glomerular filtration rate

The patient was diagnosed with CCE. Treatment with prednisolone (20 mg/day) was initiated, leading to a gradual improvement in renal function, which allowed for the tapering of prednisolone. On day 103 post MT, the patient’s BUN level, Cre level, and eGFR decreased to 39.0 mg/dL, 2.92 mg/dL, and 17.5 mL/min/1.73 m2, respectively (Figure 7).

## Discussion

We presented the case of a patient who developed renal and dermatological complications two weeks after undergoing MT for AIS and was subsequently diagnosed with CCE via skin biopsy. Cholesterol crystal embolism is characterized by arterial embolization caused by cholesterol crystals or debris originating from atherosclerotic plaques [1,2,8,9]. Risk factors for CCE include hypertension, age >50 years, smoking, hypercholesterolemia, obesity, and diabetes mellitus [1,2,8,9]. The clinical spectrum is broad, ranging from Horner’s spots in the retina and transient ischemic attacks to cerebral infarctions, signs of intestinal ischemia, renal failure, and cutaneous manifestations such as livedo reticularis and blue toe syndrome [1,2,8,9].

Cholesterol crystal embolism should be suspected when these clinical features manifest following intravascular procedures, including endovascular therapies. Differential diagnoses of CCE include contrast-induced acute kidney injury, ischemic acute tubular necrosis, drug-induced interstitial nephritis, endocarditis, aortic dissection, left atrial myxoma, lymphoma, tuberculosis, secondary syphilis, pheochromocytoma, Raynaud’s phenomenon, vasculitis, cryoglobulinemia, antiphospholipid syndrome, polycythemia vera, and thrombotic thrombocytopenic purpura [2]. Although blue toe syndrome is a hallmark of CCE, it can also occur in patients with vasculitis or endocarditis [2]. A definitive diagnosis of CCE requires histopathological confirmation, with typical findings including “ghosts” of cholesterol crystals or clefts in arterioles formed by the dissolution of cholesterol during tissue processing [2]. Symptom onset can range from one day to three months after the precipitating event [8]. Although no standardized treatments have been established, management strategies such as anticoagulant discontinuation, corticosteroids, low-density lipoprotein apheresis, plasma exchange, and statin therapy have been reported [1-4]. The prognosis of CCE is generally poor, with a reported mortality rate of 63%, increasing to 80% in histopathologically confirmed cases [8,9].

Several case reports have identified cholesterol crystals in thrombi retrieved during MT for previously undiagnosed strokes, leading to diagnoses of atheroembolic stroke [13-15]. Atherothrombotic cerebral embolism is occasionally misdiagnosed as cardiogenic cerebral embolism [15]. To the best of our knowledge, this is the first documented case of CCE occurring after MT for AIS. Previous reports of CCE following carotid artery stenting suggest an incidence rate of 0.2% [10], whereas the incidence following cardiac catheterization is reported to be 1.4% [11]. The incidence of CCE post MT remains unknown. Mechanical thrombectomy for AIS is frequently performed in patients with multiple atherosclerotic risk factors, many of whom are elderly and require urgent intervention, limiting opportunities for preventive measures such as smoking cessation and statin therapy. Additionally, the need for rapid procedural execution increases the likelihood of vascular injury, which may contribute to a higher incidence of CCE.

The rarity of CCE reports following MT may be attributed to several factors: the delayed onset of CCE after hospital discharge, which can lead to a lack of awareness among the physicians who performed the MT; the frequent co-occurrence of atrial fibrillation, resulting in the misattribution of the embolism to that condition; and the presence of multiple atherosclerotic risk factors, which may lead to misdiagnosis as atherosclerotic renal injury or peripheral artery disease. Furthermore, in the absence of lower limb symptoms, contrast-induced nephropathy may be considered. Anticoagulation is commonly initiated when the embolism is presumed to be of atrial fibrillation origin; however, this poses a significant risk in the context of CCE [1,2,8,9]. Therefore, an accurate diagnosis is critical, and CCE should be included in the differential diagnosis when renal dysfunction or lower limb ischemia occurs post MT. Diagnostic confirmation through skin biopsy or other definitive methods is essential.

## Conclusions

This case highlights the rare but significant complication of CCE following MT for AIS. The development of CCE, manifesting as blue toe syndrome and acute kidney injury, underscores the importance of maintaining clinical vigilance, especially in patients with underlying atherosclerosis and multiple risk factors. Early recognition and appropriate management can lead to partial recovery of renal function. Further research is necessary to better understand the incidence and optimal treatment strategies for CCE following MT.
